# Identifying brain effective connectivity patterns from EEG: performance of Granger Causality, DTF, PDC and PSI on simulated data

**DOI:** 10.1186/1471-2202-12-S1-P141

**Published:** 2011-07-18

**Authors:** Stefan Haufe, Vadim Nikulin, Guido Nolte

**Affiliations:** 1 Berlin Institute of Technology, Franklinstr. 28/29, D-10587 Berlin, Germany; 2 Bernstein Focus Neurotechnology, Berlin, Germany; 3 Charité University Medicine, Berlin, Germany; 4 Fraunhofer Institute FIRST, Berlin, Germany

## Background

High temporal resolution and relatively low cost make electroencephalography (EEG) the most suitable noninvasive tool for studying brain dynamics. One of the major challenges is the determination of effective connectivity, i.e., directed (causal) information flow between brain areas. The common definition of effective connectivity is based on Granger's argument, that “the cause must precede the effect” [1], which is implemented by the original Granger Causality (GC) score [1], the Directed Transfer Function (DTF) [3], Partial Directed Coherence (PDC) [2] and the Phase-slope Index (PSI) [4], all of which have been applied to EEG data previously. However, due to volume conduction in the head, the original causally-related sources are mixed into EEG channels. We conducted a realistic simulation study to investigate how volume conduction affects EEG sensor-space effective connectivity estimation.

## Methods

Two interacting sources were simulated by means of a bivariate autoregressive (AR) model with all-zero coefficients **A**_1,2_(p), but nonzero coefficients **A**_2,1_(p). The source time courses were mapped to 59 EEG sensors using the realistic spread of two dipoles with tangential orientation located 3cm below electrodes C3 and C4. Brain noise was simulated at ten random brain locations using univariate AR models, projected to the EEG and added to the signal, along with white sensor noise (SNR=1). One-hundred experiments were conducted using different sensor noise realizations, brain noise dipole locations and orientations, as well as AR coefficient realizations and innovation terms. Significance was assessed using Student's t-test.

## Results

Only PSI correctly indicated significant inter-hemispheric information flow. GC instead indicated significant symmetric bilateral flow from EEG channels with strong contributions from any of the two sources (and hence high SNR) to channels with low contributions (SNR). The estimated connectivity pattern according to DTF and PDC was exactly opposite to that of GC, i.e., information was estimated to flow from low-SNR channels to high-SNR channels. The flow reversed (i.e., resembled that of GC) when the EEG time series were normalized to unit variance. PSI and GC estimates were not affected by the normalization.

## Discussion

GC, DTF and PDC are based on the consideration that knowledge of the “driver's” past increases the prediction of the “receiver's” present state, compared to only using the receiver's past. In the presence of volume conduction, however, all EEG channels mutually “drive” each other in this respect. GC, which is based on comparing prediction errors, then determines the channel with higher SNR as the “effective” driver, since it contains more predictive information. DTF and PDC are derived from coefficients of a multivariate autoregressive (MVAR) model, which additionally depend on the scaling of the data. Interestingly, this scaling dependency is sufficient to yield significant spurious information flow even from low-variance to high-variance temporally and spatially white noise channels. PSI utilizes the imaginary part of coherence, which is provenly unaffected by (mixed) non-interacting signals (such as white noise, brain noise). The determination of effective drivers and receivers here solely depends on the *relative* contribution of the two interacting sources in each channel pair.
